# SuperAging is associated with reduced age‐related cortical neuroinflammation in the absence of neuropathology

**DOI:** 10.1002/alz70855_102354

**Published:** 2025-12-23

**Authors:** Karolina J Senkow, Allegra Kawles, Maxwell J Schleck, Caroline M Nelson, Grace Minogue, Rachel M Keszycki, Antonia Zouridakis, Alyssa Macomber, Molly A Mather, Rudolph J Castellani, Changiz Geula, Marsel Mesulam, G.R. Scott Budinger, Alexander V Misharin, Tamar Gefen, Rogan A Grant

**Affiliations:** ^1^ Northwestern University Feinberg School of Medicine, Chicago, IL, USA; ^2^ Mesulam Center for Cognitive Neurology & Alzheimer's Disease, Chicago, IL, USA; ^3^ Northwestern ADC Neuropathology Core, Northwestern University Feinberg School of Medicine, Chicago, IL, USA

## Abstract

**Background:**

It is well‐established that average or “normal” aging is associated with a gradual decline of memory capacity. SuperAgers are defined as unique individuals ≥80 years of age who show exceptional memory at least as good as individuals 20‐30 years their junior. The goal of this study was to identify the cellular and molecular substrates driving the vulnerability or preservation of memory during human aging.

**Method:**

We performed imaging spatial transcriptomics (Xenium, 10X Genomics) using a custom panel of 381 genes on paraffin‐embedded cortex (middle frontal gyrus) for the unbiased identification of more than 600,000 high‐quality cells encompassing multiple subtypes and cell states of neurons, glia, and endothelial cells with subcellular resolution. Brain specimens were obtained through the Northwestern University Alzheimer's Disease Research Center brain bank from the following individuals: 1) SuperAgers with intermediate Alzheimer's disease neuropathic change (ADNC; n = 2), or with only mild primary age‐related tauopathy (PART; n = 2) ; 2) Cognitively‐healthy individuals without pathology (*n* =  2); and 3) individuals with clinical amnestic dementia and significant ADNC or ADNC + limbic‐predominant age‐related TDP‐43 encephalopathy (LATE‐NC), stage 2 (*n* =  4).

**Result:**

We found that, in the cortex of individuals exhibiting significant postmortem neuropathology, key pro‐inflammatory glial populations emerged, including disease‐associated microglia (DAMs), oligodendrocytes characterized by NF‐κB‐responsive gene expression, and reactive astrocytes characterized by elevated expression of the monocyte and lymphocyte chemoattractant *CCL2*. In SuperAgers lacking significant neuropathology at autopsy, however, these pro‐inflammatory glial subpopulations were substantially less prevalent. Abundance of these populations was also reduced relative to “normal” agers.

**Conclusion:**

These preliminary findings suggest that the SuperAging phenotype may be associated with reduced cortical neuroinflammation at the molecular and cellular level. Further, we established a novel imaging spatial transcriptomic atlas of the aging human middle frontal gyrus across abnormal and supranormal trajectories of cognitive aging.

